# Regulatory Networks of lncRNAs, miRNAs, and mRNAs in Response to Heat Stress in Wheat (*Triticum Aestivum* L.): An Integrated Analysis

**DOI:** 10.1155/2023/1774764

**Published:** 2023-03-30

**Authors:** Dwijesh Chandra Mishra, Sayanti Guha Majumdar, Anuj Kumar, Jyotika Bhati, K. K. Chaturvedi, Ranjeet Ranjan Kumar, Suneha Goswami, Anil Rai, Neeraj Budhlakoti

**Affiliations:** ^1^ICAR-Indian Agricultural Statistics Research Institute, New Delhi, India; ^2^ICAR-Indian Agricultural Research Institute, New Delhi, India

## Abstract

Climate change has become a major source of concern, particularly in agriculture, because it has a significant impact on the production of economically important crops such as wheat, rice, and maize. In the present study, an attempt has been made to identify differentially expressed heat stress-responsive long non-coding RNAs (lncRNAs) in the wheat genome using publicly available wheat transcriptome data (24 SRAs) representing two conditions, namely, control and heat-stressed. A total of 10,965 lncRNAs have been identified and, among them, 153, 143, and 211 differentially expressed transcripts have been found under 0 DAT, 1 DAT, and 4 DAT heat-stress conditions, respectively. Target prediction analysis revealed that 4098 lncRNAs were targeted by 119 different miRNA responses to a plethora of environmental stresses, including heat stress. A total of 171 hub genes had 204 SSRs (simple sequence repeats), and a set of target sequences had SNP potential as well. Furthermore, gene ontology analysis revealed that the majority of the discovered lncRNAs are engaged in a variety of cellular and biological processes related to heat stress responses. Furthermore, the modeled three-dimensional (3D) structures of hub genes encoding proteins, which had an appropriate range of similarity with solved structures, provided information on their structural roles. The current study reveals many elements of gene expression regulation in wheat under heat stress, paving the way for the development of improved climate-resilient wheat cultivars.

## 1. Introduction

With the continuous change in climatic conditions, the rise in global temperature is a major threat to agriculture production as it impacts global food demand and security. This increasing temperature affects plant growth and development processes negatively. Heat stress is considered to be major abiotic stress which can affect the yield performance of economically important crops, including wheat (*Triticum aestivum* L.) [1, 2]. Wheat is a largely consumed cereal crop in the family Poaceae. Furthermore, wheat is the largest contributor, with nearly 30% of the world grain production and 50% of the world grain trade [3, 4]. The Food and Agriculture Organization (FAO) (https://www.fao.org/home/en) estimated that by 2050, the world would require an additional 198 million tonnes of wheat to secure food security goals, for which wheat production needs to be increased by up to 77% in the developing countries [5, 6]. The temperature anomaly distribution, on the other hand, is shifting toward higher temperatures [7], and wheat production is estimated to be reduced by up to 6% for each 1°C rise in temperature. Arenas-M et al. [8] have reported a significant reduction in grain weight (23.9%) and grain dimensions due to heat-stress in wheat. In comparison to the control condition, grain quality was also significantly impacted, with a fall in starch content (20.8%) and an increase in grain protein levels (14.6%) [8].

Plants employ different types of mechanisms at the biochemical or molecular level to cope with heat stress [9]. By activating almost all “heat shock genes” (HSGs), heat stress transcription factors (HSFs) regulate crucial parts of the heat stress response, hence defending against it. Heat shock proteins (HSPs), which prevent intracellular proteins from denaturation, are encoded by a number of heat-inducible genes known as HSGs that are up-regulated in response to heat stress. Transcription of HSG is started in response to heat by conserved heat shock elements (HSEs) in the promoter region. Plant HSFs are a complex gene family which play a crucial role in regulating transcription under heat stress [10]. To deal with heat-stress conditions, plants have a variety of adaptation, avoidance, or acclimatization strategies. Major tolerance mechanisms are also triggered to counteract stress-induced biochemical and physiological changes. These mechanisms make use of proteins, ion transporters, osmoprotectants, antioxidants, and other elements involved in signaling cascades and transcriptional regulation [11]. The excessive production of reactive oxygen species (ROS), which causes oxidative stress, is one of the main adverse effects of heat stress [12]. Heat stress alters the expression of genes, namely, transporters, regulatory proteins, detoxifying enzymes, and osmoprotectants, involved in direct protection from heat stress at the molecular level [13, 14]. In situations like heat stress, altering physiological and biochemical processes through changes in gene expression results in the progressive development of heat tolerance in the form of acclimation or, in the best-case scenario, adaptation [15, 16]. As evident from previous studies, long noncoding RNAs (lncRNAs) have a principal role in the regulatory mechanisms of plants during response to heat stress, which are highly heterogeneous, having lengths ≥200 nucleotides [17]. Over the past decade, significant progress in next-generation sequencing (NGS)-based methods has accelerated the identification and functional characterization of lncRNAs in different crop plants. lncRNAs are a class of regulatory RNAs and are well known for their pivotal role in a plethora of biological processes, including development, genomic imprinting, cell differentiation, chromatin remodeling, transcriptional activation, transcriptional interference, regulation of protein re-localization, and cell cycle at transcriptional, post-transcriptional, and post-translational levels [18]. These lncRNAs shared similarities with coding mRNA in many aspects, including splicing, polyadenylation, conserved sequences, and being transcribed by RNA Pol II [19–21]. LncRNAs can act as a precursor of microRNAs (miRNAs) and can also regulate the functions of miRNAs by acting as target mimics or decoys in both plants and animals. It also inhibits the interaction between miRNAs and their target mRNAs, thus regulating the expression of corresponding mRNAs [22, 23]. In plants, the function of most lncRNAs is still unclear as they are comparatively less explored in comparison to animal species. However, with significant development in the scientific knowledge of plant genomes, lncRNAs have been identified and characterized in different plant species, including *Arabidopsis thaliana* L., *Oryza sativa* L., *Zea mays* L., *Cucumis sativus* L., *Populus trichocarpa* L., and so on ([24], [25, 26], [27]). In earlier studies, heat stress-responsive lncRNAs have been identified and annotated in wheat [21, 28]. Despite the significant importance of lncRNAs in response to heat stress, to the best of our knowledge, there is no single report available on deciphering the regulatory network of lncRNA-miRNA-mRNA in relation to wheat. In the present study, we have identified and annotated a set of lncRNAs involved in heat stress using the publicly available wheat transcriptome data (24 SRAs) representing two conditions, namely, control and heat stressed. We also developed an RNA interaction network of miRNAs that could potentially interact with the identified lncRNA and their target genes followed by gene ontology (GO) based annotations and also mined the gene-specific SSRs and SNPs. Furthermore, three-dimensional (3D) structures of the lncRNA-targeted proteins were modeled using the homology approach.

The current study sets the framework for a better understanding of how potential lncRNAs respond to heat stress in wheat as well as provides some important insights into the genetic and structural characteristics of lncRNAs. Our analysis provides a comprehensive picture of how lncRNAs are expressed and controlled when wheat responds to heat stress. This could be used as an alternative resource to develop new wheat cultivars and increase crop yield.

## 2. Materials and Methods

### 2.1. RNA-Seq Data Used for Identification of lncRNA in Wheat

We downloaded and extracted RNAseq data related to heat stress in FastQ format from NCBI's Sequence Read Archive (SRA) database [29] (https://www.ncbi.nlm.nih.gov/sra) to identify lncRNAs responsive to heat stress in wheat. A total of 24 data files have been downloaded, which include various combinations of both control and heat-stress data at different time points, that is, 0, 1, and 4 days after treatment (DAT). The data used here belongs to leaf tissue and it was collected from all heat-stressed and controlled individual plants immediately at the end of the stress period, that is, 0, 1, and 4 DAT with 4 biological replicates [30]. Further details of the data are provided in Table 1.

### 2.2. Bioinformatics Pipeline for Identification of lncRNA

**Figure 1:**
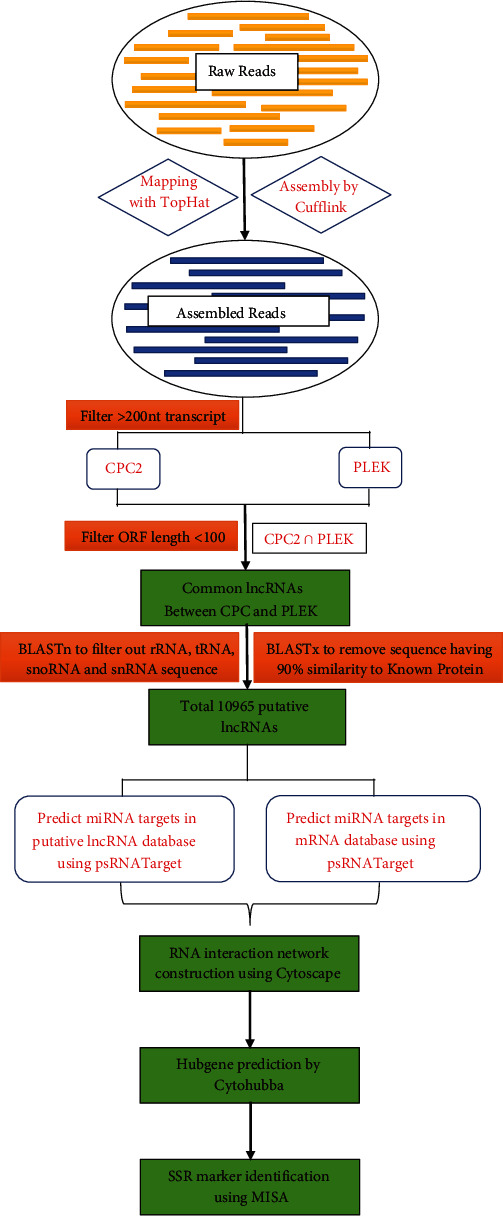
Computational pipeline for systematic identification of lncRNAs and their targets in the current study.

Initially, the FastQC tool (Andrews, 2010) was employed to check the quality of data available to us. Furthermore, low-quality sequences were trimmed using Trimmomatic [31]. Then, raw data files were mapped to the wheat reference genome (GCA_900519105.1) using the TopHat and Cufflinks software suite [32, 33]. Transcripts less than 200 nucleotides in length are filtered using an in-house Perl script. Furthermore, to avoid any potential protein-coding transcripts, the Protein-Coding Calculator2 (CPC2) tool [34] and PLEK tool (https://sourceforge.net/projects/plek/) were utilized and further filtered out the transcripts which had an open reading frame (ORF) of more than 100 amino acids. After that, the common transcripts that were predicted as noncoding by both the CPC2 and PLEK tools were selected. BLASTn was performed against the tRNA, rRNA, snRNA, and snoRNA databases, and BLASTx against the NCBI nr protein database to remove the housekeeping RNAs, and transcripts having coding potentials, respectively. Further downstream, the Augustus tool [35] was used to predict the number of exons within putative lncRNAs. These putative lncRNAs were then annotated using the Blast2GO pipeline [36]. Furthermore, the differential expressions of the transcripts were analyzed using the Cuffdiff module of the Cufflink software [37]. The results of the differential expression data were visualized by the R package CummeRbund. The whole bioinformatics procedure utilized in the current study is described in Figure 1.

### 2.3. Prediction of lncRNA Targets and Interaction Network

The psRNATarget web-server [38] was employed to predict the potential targets of identified lncRNAs in the current study. First, the targets of wheat miRNAs were predicted separately for the lncRNA database and the mRNA database of wheat. For this purpose, 119 miRNAs from wheat were selected as query sequences and a set of 10,965 predicted lncRNAs was used as a database for target prediction. Furthermore, the same set of 119 miRNAs was used as a query, and mRNA sequences of wheat available to us were used as a database for target prediction.

To build an RNA interaction network, miRNAs, their target lncRNA, and corresponding target accessibility scores provided by psRNATarget were used as input to Cytoscape software [39]. Likewise, an interaction network is also built for mRNA and miRNA separately. To get the complete interaction network of miRNA-lncRNA-mRNA, the *Merge Network* option provided in Cytoscape was used to merge these miRNA-lncRNA and miRNA-mRNA networks. A total of 101 interconnected clusters were present in the network. Furthermore, from each cluster, hub genes were identified by using the application cytoHubba, which is itself a part of the Cytoscape tool. The top 10 hub genes were identified based on 11 different algorithms, namely MCC, DMNC, MNC, Degree, EPC, BottleNeck, EcCentricity, Closeness, Radiality, Betweenness, Stress, and Clustering Coefficient. Thus, 11 sets of the top 10 hub genes were identified for one cluster, and finally, as a consensus, the “union” of these 11 sets of hub genes was taken as the final set of hub genes for the same cluster. In the same way, hub genes were found for each of the 101 clusters that were found in this study.

### 2.4. Structure Modeling of Target Genes Encoding Proteins and Structure Evaluation

The representative mRNAs that are associated with response to heat stress in wheat (based on the Blast2GO annotation) were selected for structure modeling. Totally, 6 mRNAs, namely, JP928422.1, JP879666.1, CV762197.1, CJ542498.1, GD186945.1, and HG916218.1, were considered and their corresponding amino acid sequences were obtained using ORF Finder (https://www.ncbi.nlm.nih.gov/orffinder/) tool of NCBI. 3D structure modeling of these representative proteins was predicted by using an automated Phyre2 program [40], based on the fold recognition method of protein structure prediction. The structural quality of modeled 3D structures was investigated by calculating the Ramachandran plot using PROCHECK tool [41] and Protein Structure Verification Server (PSVS) [42]. We kept going with the loop modeling step until we had a structure with more than 90% of the residues in the most preferred region and none in the least preferred region of the Ramachandran plot.

### 2.5. Prediction of SNPs and SSRs within Targets of lncRNAs

SNPs were mined only for the selected mRNAs. To start with, first, VCF file of the wheat (GCA_900519105.1) was downloaded from the Ensembl plants database available at https://plants.ensembl.org/triticum_aestivum [43], which also includes positions of SNPs on the wheat genome. The corresponding positions of the SNPs were located by mapping the mRNA onto the reference genome. We have also used the MISA tool [44] to figure out where the SSR markers are on the hub genes.

## 3. Results and Discussion

### 3.1. Genome-Wide Identification of Heat Stress-Responsive lncRNAs

**Figure 2:**
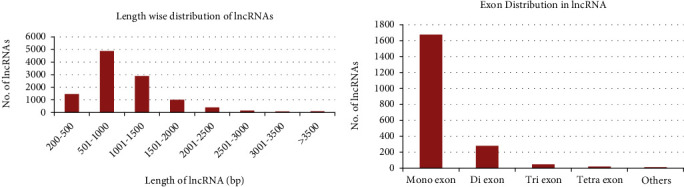
**(a and b).** The characteristic features of wheat lncRNAs.

A total of 146,865 transcripts were selected using standard threshold (e.g., length of transcript >200 nucleotides) from the whole set of merged transcripts (147029). To know the coding potential, these 146,865 transcripts were analyzed with CPC2 and PLEK tools. Further, the consensus results obtained from CPC2 and PLEK (after removing transcripts having an ORF length of >100 amino acids) were considered for further analysis (a total of 15,830 transcripts). These transcripts were searched against tRNA, rRNA, snoRNA, and snRNA databases to filter out housekeeping genes using blastn with a standard threshold i.e., *e*-value (<1 × 10^−5^) and identity (>90%). Also, these transcripts were searched against the NCBI nr protein database using BLASTx to remove any potential protein-coding transcripts. Finally, a total of 10,965 putative lncRNAs were found, with the average length of putative lncRNAs being 1046 bp. For further reference, the lengthwise distribution and exonic distribution of lncRNAs were given in Figures 2(a) and 2(b), respectively. From previous studies, it was evident that the average length of lncRNAs varies from species to species, that is, 285, 287, 463, and 323 bp for *Arabidopsis*, cluster bean, kiwifruit, and rice, respectively [45–48]. It was also observed from Figure 2(a) that almost 93% of total putative lncRNAs fall between 200 and 2000 bp lengths. In addition to that, it was observed that 2038 (18%) of 10,965 lncRNAs contain exons from the analysis performed by the Augustus tool. Here, in wheat, a total of 82% lncRNAs were mono-exonic (Figure 2(b)) as compared to the previously reported 81% in both kiwifruit and maize [45, 48] and 91% in cluster bean [47]. Besides, one lncRNA (i.e., TCONS_00042125) having a total length of 1810 bp has nine exons, which is the highest among all lncRNAs.

### 3.2. Identification of Targeted lncRNAs by Wheat miRNA and their mRNA Target

**Figure 3:**
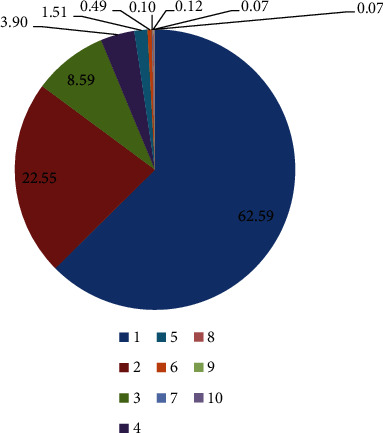
Distribution of percentage of lncRNAs targeted by 1–10 miRNAs in wheat.

From the results of psRNATarget, it was observed that out of 109,65 lncRNAs, a total of 4098 (37%) were targeted by 119 miRNAs in wheat. Interestingly, about 62.59% of lncRNAs were targeted by only one miRNA, whereas only 3 lncRNAs were targeted by 10 miRNAs each. We have filtered the results to retain only relevant target lncRNAs (having a Target Accessibility Score <20) and found only 87 suitable lncRNAs for further analysis. The details about the distribution of the percentage of lncRNAs targeted by 1–10 miRNAs in wheat are given in Figure 3. As for the targeted mRNAs, a total of 42,052 mRNAs were targeted by 119 miRNAs in wheat, among which 3170 mRNAs had a Target Accessibility Score <20.

### 3.3. lncRNA-miRNA-mRNA Interaction Network and Hub Genes Prediction

**Figure 4:**
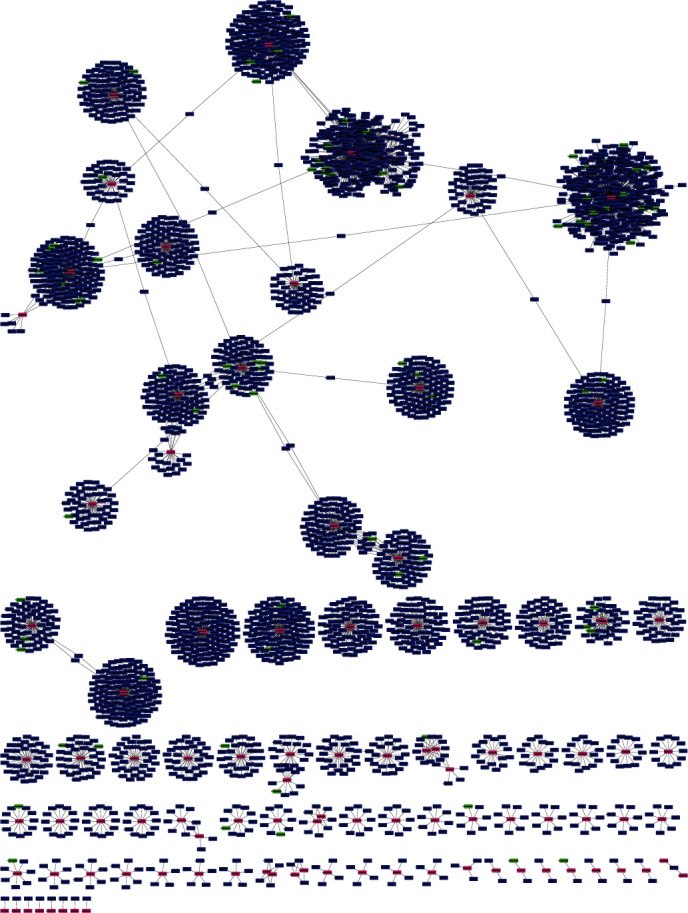
Complete lncRNA-miRNA-mRNA interaction network.

**Figure 5:**
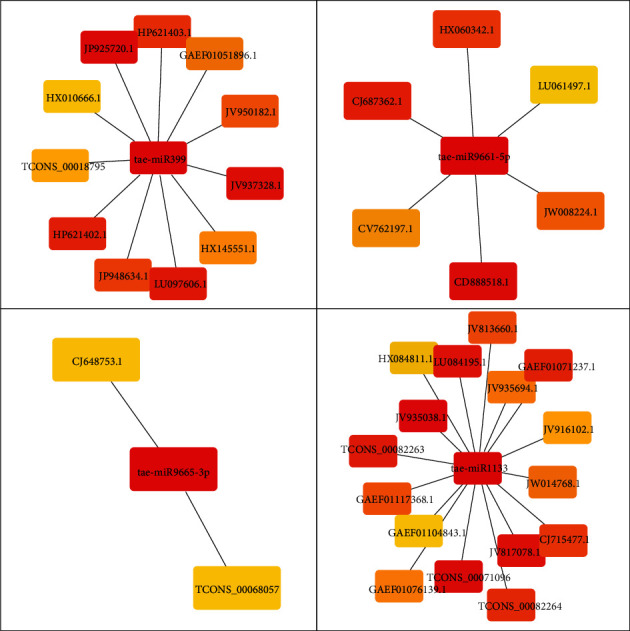
Hub genes for miRNA tae-miR399, tae-miR9661-5p, tae-miR9665-3p, and tae-miR1133.

**Figure 6:**
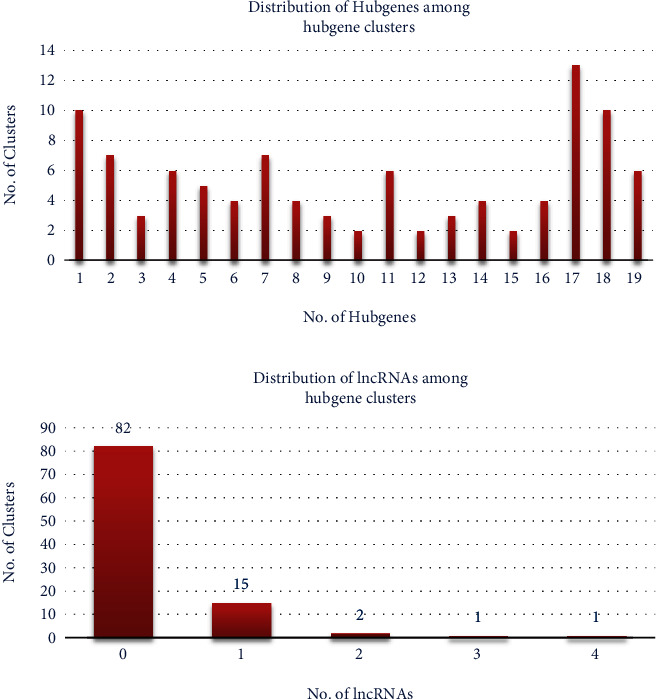
(a and b) Distribution of hub genes and lncRNAs among hub gene clusters.

A total of 87 lncRNAs and 3170 mRNAs targeted by 101 miRNAs were used to develop the interaction network by Cytoscape. The complete lncRNA-miRNA-mRNA interaction network is given in Figure 4. A total of 101 separate clusters were found for each miRNA in the network. Hub genes were predicted separately for each miRNA (Figure 5). The total number of hub genes found was 931, whereas for each cluster (or miRNA), the number of hub genes ranges between 1 and 19.  Figure 6(a) depicts the distribution of the number of hub genes among each miRNA. It was also observed that 82 miRNAs have no lncRNA targets among their hub genes, whereas 15 miRNAs have only one lncRNA target among their hub gene sequences.  Figure 6(b) depicts the distribution of the number of targeted lncRNAs among the total hub genes.

**Figure 7:**
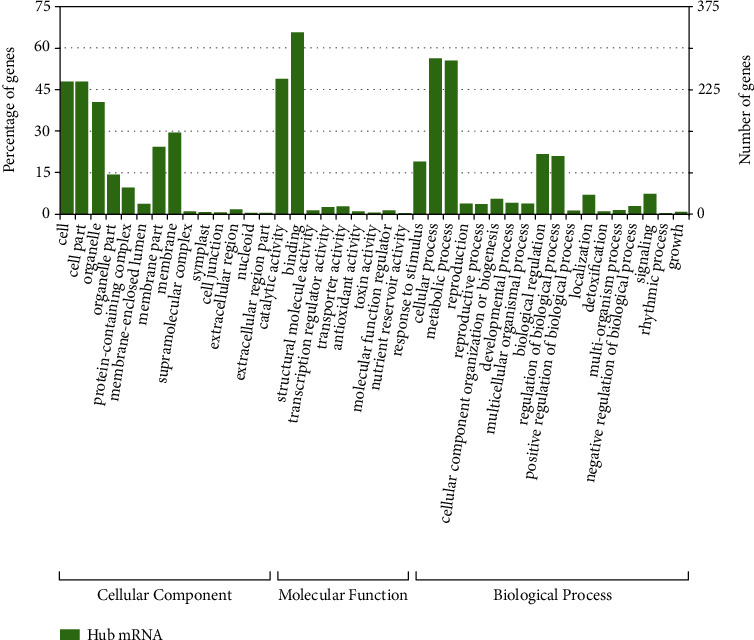
GO classification of mRNA hub genes.

The majority of hub genes were localized to cells and their parts under the cellular component category, followed by organelle. The mRNA hub genes were annotated by BLAST2GO and showed maximum genes associated with binding activity, catalytic activity under the molecular function category [49–51], and cellular process and metabolic process under the biological process category [52–54] (Figure 7).

### 3.4. Mining of Gene-Specific SSRs

**Figure 8:**
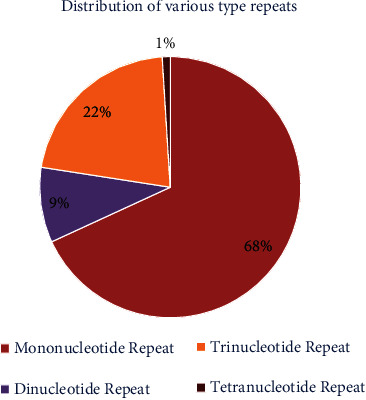
Distribution of various type repeats on hub gene sequences.

Simple sequence repeats (SSRs), also known as microsatellites, are tandemly repeated DNA sequences that consist of one to six nucleotide units. SSRs have distinct qualities, such as co-dominant inheritance, extensive genome coverage, high abundance, high reproducibility, and multi-allelic nature. Due to these characteristics, they are the most widely recognized genetic markers actively used in plant breeding. SSR markers are widely utilized for a variety of purposes, including population genetics, functional diversity, linkage mapping, DNA fingerprinting, and most importantly, assisted breeding techniques [55]. A total of 931 numbers of hub gene sequences, which include both lncRNA and mRNA targets of miRNAs, were analyzed by the MISA tool. Among them, only 171 sequences were found to have SSR markers on them. The total number of identified SSRs was 204, whereas 26 sequences having more than 1 SSR were found. Among 171 sequences, only 3 were putative lncRNAs and the remaining were mRNAs of wheat.  Table 2 consists of all types of SSRs and the number of their occurrences in the hub gene sequences. The distribution of various types of repeats (viz. mononucleotide, dinucleotide, trinucleotide, and tetranucleotide) is given in Figure 8. The mined SSRs from 931 hub gene sequences are provided in Supplementary Table 1. It is evident from previous studies that SSR markers are associated with heat-stress-responsive traits [56–58]. Sharma et al. have reported the discovery of a total of 182 alleles by assaying 52 SSRs on 40 genotypes of bread wheat [59]. In the present study, we have mined a total of 204 SSR markers, which can distinguish between heat-tolerant and heat-susceptible wheat genotypes, which will be useful in breeding programs. Breeders will be able to choose heat-tolerant wheat genotypes by using polymorphic SSR markers or marker-assisted selection in the early stages of growth.

### 3.5. Protein Structure Prediction and Structure Verification

**Figure 9:**
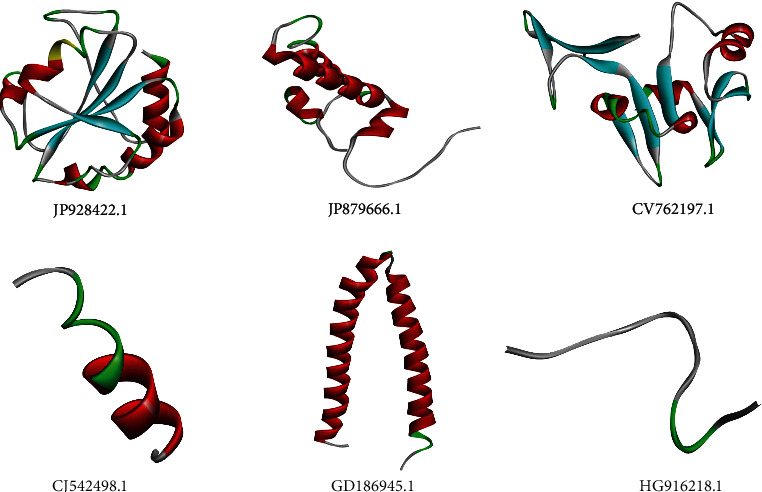
Predicted 3D structure models of heat-responsive representative proteins. Protein structures are visualized using Discovery Studio programs.

**Figure 10:**
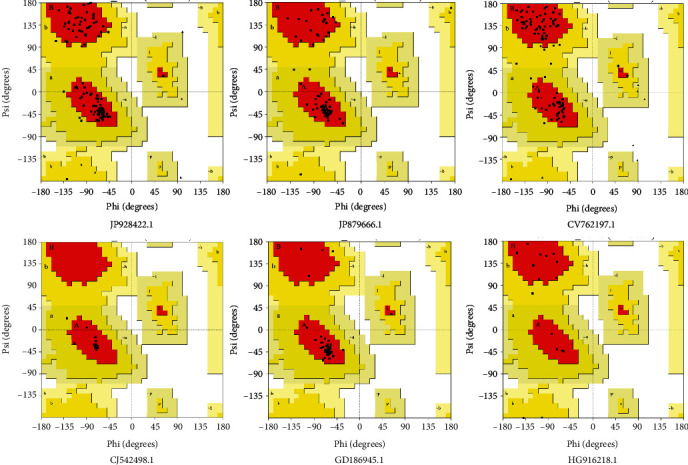
Calculated Ramachandran plots of modeled 3D structures using PSVS server.

Protein 3D structures of six representative proteins were predicted using the Phyre2 protein fold recognition server. Template structure PDB ID, sequence alignment scores, confidence values, resolution, template chain, and description are displayed in Supplementary Table 2. During modeling, template structures were selected based on sequence alignment between target and template structures. Sequence alignment between template and target indicated an acceptable range of similarity (94%), which is the confirmation of previous studies reported on the 3D structure models of different proteins [5, 60, 61]. Protein structures predicted using the Phyre server were further visualized in different chemical shapes using Discovery studio programs as shown in Figure 9. Modeled structure showed <1 Å RMSD with homolog template structures as a result of superposition and comparison of protein structures. The structure quality of modeled 3D structures was evaluated with the prediction of Ramachandran plots through the calculation of phi (*Φ*) and psi (*ψ*) torsion angles. As evident from Figure 10, calculated Ramachandran plots showed up to 96.7% of residues falling in the most favored regions (Table 3). As per the general rule, a good protein model must have over 90% of its residues in the most favored regions [62]. Predicted structure models can be utilized for structure-based functional annotation of these heat-responsive proteins. The SNPs located on these six mRNAs were also identified (Table 4). Plants under high temperature stress produce more HSPs and less of their usual proteins. As shown in Table 4, majorly 6 genes showed direct homology with HSPs. HSPs act as chaperones regulating the folding, accumulation, localization, and degradation of normal proteins and combating the damaging effects of heat and other stress on plants [63–65]. Mishra et al. [66] have also reported 6 genes (viz., HSFA6e, HSP90, HSP17, MAPK, CDPK, and SOD) associated to heat stress in wheat. Among which, HSP17 has shown manifold change in their expression in heat stress as compared to the control condition [66]. HSP21 was discovered to bind with the plastid nucleoid protein pTAC5, and it was shown to be essential for the growth of chloroplasts in *Arabidopsis* under heat stress [67]. The rate of carbon assimilation under sudden heat shock (SHS) stress and the small HSPs amplification levels were shown to be strongly correlated in a study on maize [68].

### 3.6. Differentially Expressed Genes and lncRNAs in Heat-Stress Conditions

**Figure 11:**
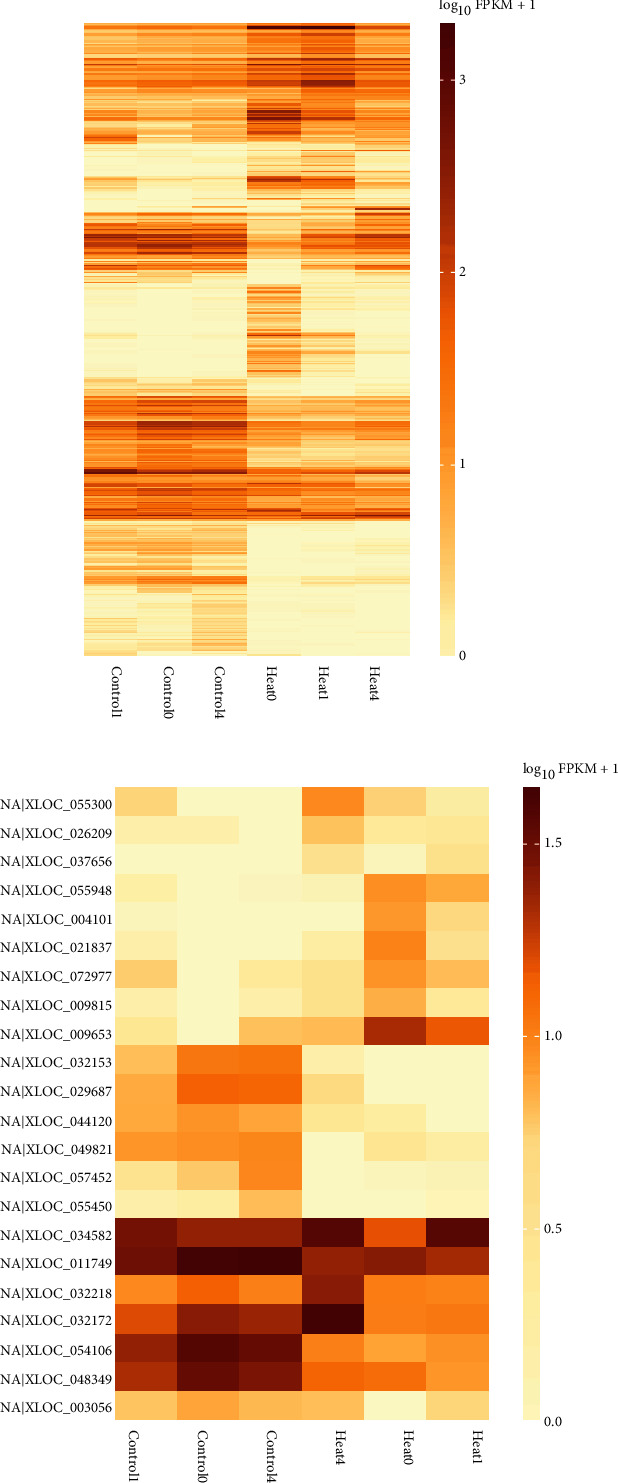
(a) Heatmap of differentially expressed transcripts in all the samples; (b) heatmap of differentially expressed putative lncRNAs in all the samples.

A total of 153, 143, and 211 differentially expressed transcripts were found under 0 DAT, 1 DAT, and 4 DAT conditions of heat stress, respectively. Among them, 18 transcripts were differentially expressed both under 0 DAT and 1 DAT conditions, while 25 transcripts were differentially expressed under both 1 DAT and 4 DAT conditions. It was also found that there were 13 common transcripts which were differentially expressed under both the 0 DAT and 4 DAT conditions. Only four transcripts (viz., TCONS_00034685, TCONS_00074321, TCONS_00103626, and TCONS_00145241) were found to be differentially expressed in all three conditions. The heat map of the differentially expressed transcripts is given in Figure 11. It is also observed that among 153 differentially expressed transcripts under the 0 DAT condition, 9 were putative lncRNAs. Whereas, among 143 and 211 differentially expressed transcripts, 2 and 11 putative lncRNAs were found under 1 DAT and 4 DAT conditions, respectively.

Out of these 9 lncRNAs under the 0 DAT condition, 3 lncRNAs, namely, TCONS_00008075, TCONS_00018609, and TCONS_00105378 were differentially expressed and mapped to 48 GO terms about the biological process, molecular function, and cellular component. Similarly, under 1 DAT and 4 DAT conditions, 1 i.e., TCONS_00071103 (115 GO terms) and 3 lncRNAs i.e., TCONS_00049265, TCONS_00064961, and TCONS_00104542 (7 GO terms) were differentially expressed, respectively. The GO term categorized for the biological process GO:0055114 was prominently found under 0 DAT and 1 DAT conditions, confirming the oxidation-reduction process which is central to both genetic and epigenetic control of plant responses to heat stress [69, 70]. The other noticeable GO terms under the biological process were GO:0006355, GO:0016310, and GO:0006468, associated with the regulation of transcription [71, 72], phosphorylation, and protein phosphorylation [73, 74], which showed regulating heat stress in plants.

For the cellular component category, the predominant GO terms were GO:0016020, GO:0016021, and GO:0005634, followed by GO:0005886. GO: 0016020 and GO: 0016021 demonstrated membrane localization and its integral components. The rise in temperature changes the membrane properties, ranging from their fluidity and permeability, which alters the lipid composition and might affect the lipid and membrane proteins' interactions [75]. GO:0005634 showed cellular localization to the nucleus, while GO:0005886 to the plasma membrane. Heat stress increases the degree of oxidation of the nucleus and cytosol (GO:0005737) as noticed in *Arabidopsis* leaf epidermal and stomatal guard cells [70].

The maximum occurrence of GO:0046872 terms about metal ion binding activity clearly shows their role in the regulation of stress response, gene expression, and their regulation along with posttranslational modifications and cell signaling [76]. The GO terms, namely, GO:0004497 and GO:0016740 showed molecular function associated with monooxygenase activity and transferase activity [71], respectively. The plant cytochrome P450/75B genes showing monooxygenase activity were highly up-regulated under heat stress [77, 78].

### 3.7. Functional Annotation of lncRNAs

A total of 3288 (30%) putative lncRNAs out of 10965 were annotated by Blast2GO. The similarity search of identified lncRNA showed the highest similarity with gamma, delta, and omega gliadin genes and LMW HSPs. Wang and co-workers [79] summarized those abiotic stresses that induce complex proteomic changes in wheat grains ranging from the up-regulation of processes that are required for stress adaptation and tolerance, which is accompanied by kernel weight reduction. The studies also showed that heat stress increases the amount of alpha and gamma gliadin during the flowering or post-anthesis stage. The alpha-gliadin genes were found to be up-regulated in conditions of heat stress [80], while gamma and omega gliadins are down-regulated [81, 82]. The increase in heat during the growing stages of plants in general increases their overall grain protein content [83]. The HSPs are produced when eukaryotes respond to abiotic stress and act as chaperons who protect cellular proteins from damage during the stress. These HSPs are regulated by transcription factors known as Heat Shock Factors (HSFs). These HSFs combat various stress conditions and are present in abundance in plants [84].

**Figure 12:**
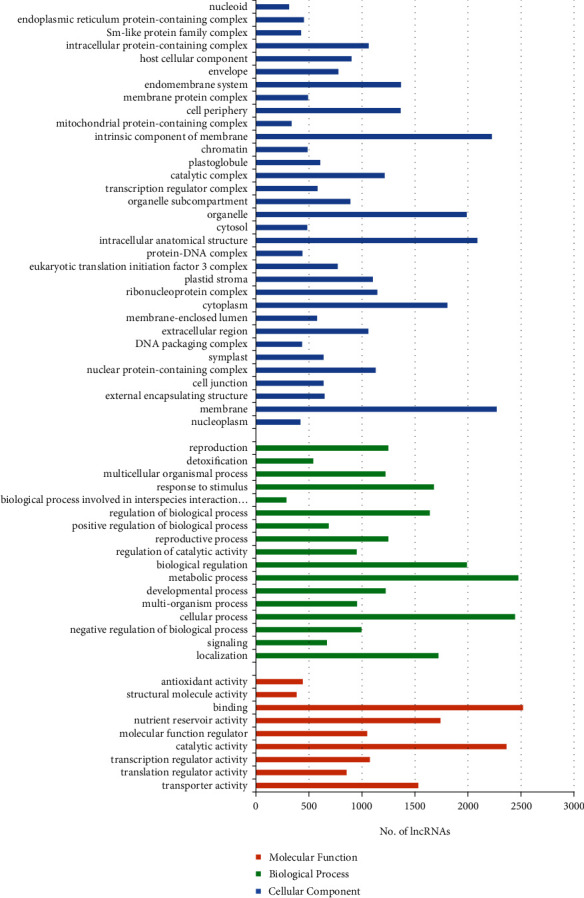
*In silico* functional annotation of putative lncRNAs.

Figure 12 represents the number of transcripts (lncRNAs) involved in the three components (viz., “biological process,” “cellular components,” and “molecular functions”) of GO. As shown in Figure 12, the most enriched terms for “biological process” were “metabolic process” and “cellular process”. In the case of “molecular function,” most enriched terms were “binding” and “catalytic activity” [85, 86], whereas “membrane” and “intrinsic component of membrane” were the most enriched terms for “cellular components” [68]. GO enrichment analysis suggested that the lncRNAs under heat stress were primarily involved in metabolic processes and cellular processes along with regulation of biological activity and response to stimulus [87–89]. These lncRNAs are widely involved in metabolic and cellular processes [90]. Heat stress affects the overall growth and development of plants along with an increase in oxidative damage, decreases photosynthesis and water retention, and finally, decreases yield. These lncRNAs regulate the activity of transcription factors and their associated genes [91]. lncRNAs play a major role in regulating biological activity mainly epigenetic activity [92, 93] as shown in cucumber [94], *Arabidopsis* [91]. These are also associated with plant hormone signal transduction pathways in response to heat stress [95]. A few lncRNAs also showed involvement in the transposable activity [96].

## 4. Conclusion

In this study, 10,965 lncRNAs were screened and identified *in silico* using RNAseq data related to heat stress in wheat (*Triticum aestivum*). Heat stress-related differentially expressed lncRNAs were also identified at three time points. To build an RNA interaction network, the predicted lncRNAs and their related protein-coding heat stress-responsive target genes and miRNAs were identified. This study shows the characteristics and expression patterns of lncRNAs during heat stress at various time periods, which may be used to investigate molecular regulation of heat-stress sensitive genes subject to the wet lab validation of results obtained in the current study. Given the need for cereals, these predicted resources might be used to investigate the biological causes of heat stress responses, provided they are validated in a wet lab. The interaction of lncRNAs with coding sequences will result in the formation of new regulatory networks that may be used for crop improvement. As reference genomes have become more widely available, our understanding of lncRNAs has evolved. This has led to the development of better cultivars to feed the world's growing population.

## Figures and Tables

**Table 1:** Data description.

Data description	Accession number	Time points	Tissue
4_DAT_Heat	SRX3963094, SRX3963093, SRX3963092, SRX3963091	4 days (heat-stressed individuals)	Leaf
4_DAT_Control	SRX3963090, SRX3963089, SRX3963088, SRX3963087	4 days (controlled individuals)	Leaf
1_DAT_Heat	SRX3963086, SRX3963085, SRX3963084, SRX3963083	1 day (heat-stressed individuals)	Leaf
1_DAT_Control	SRX3963082, SRX3963081, SRX3963080, SRX3963079	1 day (controlled individuals)	Leaf
0_DAT_Heat	SRX3963078, SRX3963077, SRX3963076, SRX3963075	0 day (heat-stressed individuals)	Leaf
0_DAT_Control	SRX3963074, SRX3963073, SRX3963072, SRX3963071	0 day (controlled individuals)	Leaf

**Table 2:** Different types of SSRs and the number of their occurrence.

Sl. no.	Repeats	Total	Sl. no.	Repeats	Total
1	A/T	128	9	ACC/GGT	4
2	C/G	11	10	ACG/CGT	1
3	AC/GT	7	11	AGC/CTG	7
4	AG/CT	8	12	AGG/CCT	4
5	AT/AT	3	13	ATC/ATG	2
6	CG/CG	1	14	CCG/CGG	17
7	AAC/GTT	3	15	AACC/GGTT	1
8	AAG/CTT	6	16	AATT/AATT	1

**Table 3:** Statistics of calculated Ramachandran plot of representative proteins.

Parameters	JP928422.1 (%)	JP879666.1 (%)	CV762197.1 (%)	CJ542498.1 (%)	GD186945.1 (%)	HG916218.1 (%)
Most favored regions	94.3	94.4	87.9	92.3	96.7	80.0
Additionally allowed regions	5.7	5.6	12.1	7.7	3.3	20.0
Generously allowed regions	0.0	0.0	0.0	0.0	0.0	0.0
Disallowed regions	0.0	0.0	0.0	0.0	0.0	0.0

**Table 4:** Number of identified SNPs on Heat stress related mRNAs.

mRNA accession no.	Chromosome no.	Number of SNPs on mRNA
CV762197.1	1A	78
CJ542498.1	7A	13
JP879666.1	2D	13
JP928422.1	4D	19
GD186945.1	2A	—
HG916218.1	1B	—

## Data Availability

The data used to support the findings of this study are available in the public domain NCBI (https://www.ncbi.nlm.nih.gov/sra/) and accession numbers are mentioned in the manuscript in Table 1.

## Supplementary Materials

Supplementary Materials Supplementary Table 1: Details of mined SSR markers from hub genes. Supplementary Table 2: Details of template protein used in structure prediction in Phyre2.
