# Supplementary material for: Regulatory Networks of lncRNAs, miRNAs, and mRNAs in Response to Heat Stress in Wheat (Triticum Aestivum L.): An Integrated Analysis
Source: Int J Genomics. 2023 Mar 30;2023:1774764. doi: 10.1155/2023/1774764 (PMC10079388; doi:10.1155/2023/1774764)
Supplement: Supplementary Materials — Supplementary Table 1: Details of mined SSR markers from hub genes. Supplementary Table 2: Details of template protein used in structure prediction in Phyre2. [file 1774764.f1.docx]

**Supplementary Table 1.** Details of mined SSR markers from hubgenes

| **ID** | **SSR nr.** | **SSR type** | **SSR** | **Size** | **Start Position** | **End Position** |
| --- | --- | --- | --- | --- | --- | --- |
| TCONS_00012884 | 1 | p2 | (GA)9 | 18 | 82 | 99 |
| TCONS_00068057 | 1 | p2 | (GT)6 | 12 | 712 | 723 |
| TCONS_00119208 | 1 | p3 | (TTG)12 | 36 | 89 | 124 |
| DR739551.1 | 1 | p1 | (A)22 | 22 | 1054 | 1075 |
| CK215494.1 | 1 | p1 | (T)29 | 29 | 19 | 47 |
| CK213337.1 | 1 | p1 | (T)15 | 15 | 20 | 34 |
| CK213203.1 | 1 | p1 | (T)21 | 21 | 24 | 44 |
| CK211362.1 | 1 | p1 | (T)16 | 16 | 23 | 38 |
| CK210798.1 | 1 | p1 | (A)15 | 15 | 528 | 542 |
| CK209656.1 | 1 | p1 | (A)15 | 15 | 249 | 263 |
| CK207533.1 | 1 | p1 | (T)15 | 15 | 16 | 30 |
| CK207533.1 | 2 | p1 | (G)10 | 10 | 281 | 290 |
| CK207485.1 | 1 | p1 | (A)14 | 14 | 1011 | 1024 |
| CK206444.1 | 1 | p3 | (GCG)5 | 15 | 192 | 206 |
| CV776184.1 | 1 | p3 | (GCG)5 | 15 | 311 | 325 |
| CV775565.1 | 1 | p3 | (CAG)7 | 21 | 346 | 366 |
| CV765922.1 | 1 | p1 | (A)15 | 15 | 729 | 743 |
| CV764487.1 | 1 | p3 | (ACC)5 | 15 | 113 | 127 |
| DR740330.1 | 1 | c | (A)11ttagattgcaaaaaan(A)12 | 39 | 907 | 945 |
| DR735126.1 | 1 | p1 | (T)56 | 56 | 123 | 178 |
| DR735126.1 | 2 | p1 | (T)10 | 10 | 403 | 412 |
| DR733919.1 | 1 | p1 | (A)24 | 24 | 870 | 893 |
| CK199352.1 | 1 | p1 | (A)15 | 15 | 743 | 757 |
| CK198314.1 | 1 | p1 | (T)42 | 42 | 118 | 159 |
| CK197689.1 | 1 | p1 | (T)27 | 27 | 118 | 144 |
| CK196934.1 | 1 | p1 | (T)18 | 18 | 116 | 133 |
| CK194250.1 | 1 | p1 | (T)25 | 25 | 119 | 143 |
| CK193704.1 | 1 | p1 | (T)17 | 17 | 117 | 133 |
| CK193704.1 | 2 | c | (G)11taaggn(G)11 | 28 | 771 | 798 |
| AL811237.1 | 1 | p1 | (T)10 | 10 | 374 | 383 |
| AL810223.1 | 1 | p3 | (AGC)6 | 18 | 462 | 479 |
| AL823135.1 | 1 | p1 | (A)10 | 10 | 207 | 216 |
| LU034004.1 | 1 | p1 | (T)16 | 16 | 10 | 25 |
| LU079899.1 | 1 | p3 | (GCG)5 | 15 | 146 | 160 |
| LU097606.1 | 1 | c | (GAG)6ca(GGC)5 | 35 | 227 | 261 |
| LU026844.1 | 1 | p1 | (T)12 | 12 | 156 | 167 |
| LU051820.1 | 1 | p1 | (G)20 | 20 | 223 | 242 |
| LU051820.1 | 2 | c | (TAAT)6aatatatataatatatttaaaaaaaatataatatttatatttaattattaattaaaaaaataaat(A)10 | 99 | 641 | 739 |
| HX155656.1 | 1 | p1 | (A)10 | 10 | 467 | 476 |
| HX160303.1 | 1 | p1 | (A)10 | 10 | 628 | 637 |
| HX101353.1 | 1 | p1 | (A)10 | 10 | 279 | 288 |
| HX122391.1 | 1 | p3 | (GCC)7 | 21 | 563 | 583 |
| HX253834.1 | 1 | p3 | (TCC)6 | 18 | 98 | 115 |
| HX176619.1 | 1 | p1 | (A)10 | 10 | 761 | 770 |
| HX152490.1 | 1 | p1 | (A)10 | 10 | 425 | 434 |
| HX151955.1 | 1 | p3 | (CGG)6 | 18 | 368 | 385 |
| HX125337.1 | 1 | p1 | (A)10 | 10 | 521 | 530 |
| HX149419.1 | 1 | p3 | (CCG)7 | 21 | 689 | 709 |
| CJ881743.1 | 1 | p1 | (A)10 | 10 | 144 | 153 |
| CJ880541.1 | 1 | p1 | (A)10 | 10 | 147 | 156 |
| CJ841562.1 | 1 | p1 | (A)10 | 10 | 65 | 74 |
| CJ834430.1 | 1 | p1 | (A)10 | 10 | 638 | 647 |
| CJ825348.1 | 1 | p1 | (A)10 | 10 | 630 | 639 |
| CJ823560.1 | 1 | p1 | (A)10 | 10 | 541 | 550 |
| CJ822171.1 | 1 | p3 | (TCA)5 | 15 | 302 | 316 |
| CJ799132.1 | 1 | p1 | (A)10 | 10 | 560 | 569 |
| CJ798528.1 | 1 | p1 | (A)10 | 10 | 434 | 443 |
| CJ905420.1 | 1 | p1 | (A)10 | 10 | 407 | 416 |
| CJ775134.1 | 1 | p1 | (A)10 | 10 | 638 | 647 |
| CJ523517.1 | 1 | p1 | (A)11 | 11 | 19 | 29 |
| CJ648753.1 | 1 | p2 | (GT)6 | 12 | 133 | 144 |
| CJ654729.1 | 1 | p1 | (A)10 | 10 | 167 | 176 |
| CJ642568.1 | 1 | p1 | (A)10 | 10 | 512 | 521 |
| CJ726575.1 | 1 | p1 | (A)10 | 10 | 684 | 693 |
| CJ715477.1 | 1 | c | (CT)6cactct(GC)6 | 30 | 124 | 153 |
| CJ653710.1 | 1 | p3 | (CGC)6 | 18 | 254 | 271 |
| CJ632118.1 | 1 | p1 | (A)10 | 10 | 322 | 331 |
| CJ600810.1 | 1 | p2 | (TA)6 | 12 | 796 | 807 |
| CJ688945.1 | 1 | p3 | (GCC)5 | 15 | 28 | 42 |
| CJ688945.1 | 2 | p2 | (GT)9 | 18 | 383 | 400 |
| CJ688945.1 | 3 | p1 | (A)10 | 10 | 603 | 612 |
| CJ608579.1 | 1 | p3 | (TCA)5 | 15 | 305 | 319 |
| CJ696765.1 | 1 | p1 | (A)10 | 10 | 620 | 629 |
| CJ724067.1 | 1 | p1 | (T)10 | 10 | 569 | 578 |
| CJ634239.1 | 1 | p1 | (A)10 | 10 | 511 | 520 |
| BJ237671.1 | 1 | p3 | (GTT)5 | 15 | 384 | 398 |
| BJ237671.1 | 2 | c | (TTG)7(CTG)5 | 36 | 625 | 660 |
| BJ303227.1 | 1 | p1 | (A)10 | 10 | 341 | 350 |
| BJ269725.1 | 1 | p1 | (A)10 | 10 | 528 | 537 |
| BJ268559.1 | 1 | c | (A)11cc(A)10 | 23 | 327 | 349 |
| JZ884753.1 | 1 | p1 | (T)11 | 11 | 418 | 428 |
| CO348675.1 | 1 | p1 | (A)18 | 18 | 427 | 444 |
| CO348445.1 | 1 | p1 | (A)21 | 21 | 453 | 473 |
| DR732899.1 | 1 | p1 | (A)10 | 10 | 1061 | 1070 |
| CN009747.1 | 1 | p1 | (A)19 | 19 | 589 | 607 |
| CK163420.1 | 1 | p2 | (GT)7 | 14 | 966 | 979 |
| CD934018.1 | 1 | p3 | (GCT)6 | 18 | 334 | 351 |
| CD913687.1 | 1 | p1 | (A)11 | 11 | 532 | 542 |
| CD866312.1 | 1 | p2 | (GA)7 | 14 | 17 | 30 |
| CD454678.1 | 1 | p1 | (T)14 | 14 | 1 | 14 |
| CD453087.1 | 1 | p1 | (T)28 | 28 | 1 | 28 |
| CA730634.1 | 1 | p3 | (GCG)5 | 15 | 117 | 131 |
| CA718138.1 | 1 | p1 | (T)10 | 10 | 1 | 10 |
| CA707726.1 | 1 | p1 | (T)14 | 14 | 1 | 14 |
| CA688285.1 | 1 | p1 | (T)35 | 35 | 1 | 35 |
| CA684033.1 | 1 | p1 | (T)13 | 13 | 1 | 13 |
| CA679118.1 | 1 | p1 | (A)12 | 12 | 360 | 371 |
| CA669630.1 | 1 | p1 | (T)27 | 27 | 38 | 64 |
| CA651375.1 | 1 | p1 | (A)12 | 12 | 186 | 197 |
| CA635778.1 | 1 | p1 | (A)12 | 12 | 451 | 462 |
| CA633244.1 | 1 | p1 | (A)12 | 12 | 604 | 615 |
| CA614252.1 | 1 | p1 | (A)12 | 12 | 453 | 464 |
| CA612943.1 | 1 | p1 | (T)29 | 29 | 1 | 29 |
| CA601522.1 | 1 | p1 | (A)12 | 12 | 321 | 332 |
| CA595092.1 | 1 | p1 | (A)12 | 12 | 256 | 267 |
| BU099671.1 | 1 | p1 | (A)23 | 23 | 667 | 689 |
| BQ607089.1 | 1 | p1 | (T)18 | 18 | 15 | 32 |
| BQ607089.1 | 2 | p3 | (ACC)5 | 15 | 277 | 291 |
| BQ161661.1 | 1 | p1 | (T)18 | 18 | 1 | 18 |
| BQ161549.1 | 1 | p1 | (T)18 | 18 | 1 | 18 |
| BE516383.1 | 1 | p1 | (T)25 | 25 | 4 | 28 |
| BE426352.1 | 1 | p1 | (T)38 | 38 | 2 | 39 |
| AK451219.1 | 1 | p1 | (T)14 | 14 | 4 | 17 |
| AK451545.1 | 1 | p3 | (AGG)7 | 21 | 464 | 484 |
| AK451545.1 | 2 | p1 | (A)14 | 14 | 3173 | 3186 |
| AK451318.1 | 1 | p1 | (A)16 | 16 | 2097 | 2112 |
| AK448211.1 | 1 | p1 | (T)11 | 11 | 3562 | 3572 |
| AK457922.1 | 1 | p1 | (A)13 | 13 | 1949 | 1961 |
| AK456629.1 | 1 | p3 | (GCG)5 | 15 | 169 | 183 |
| AK456629.1 | 2 | p1 | (A)16 | 16 | 1739 | 1754 |
| AK455047.1 | 1 | p1 | (A)15 | 15 | 1013 | 1027 |
| AK454466.1 | 1 | p1 | (A)16 | 16 | 2786 | 2801 |
| AK454427.1 | 1 | p1 | (A)16 | 16 | 2300 | 2315 |
| AK454001.1 | 1 | c | (TA)6tgtgtagtagcattatcaatgagaacaactattcactttt(A)14 | 66 | 1293 | 1358 |
| AK452887.1 | 1 | p1 | (A)18 | 18 | 2068 | 2085 |
| AK452470.1 | 1 | p1 | (A)11 | 11 | 1422 | 1432 |
| AK452470.1 | 2 | p3 | (CCG)8 | 24 | 2700 | 2723 |
| DQ512368.1 | 1 | c | (CTT)6cc(TCT)5 | 35 | 35 | 69 |
| HQ650114.1 | 1 | p3 | (CCG)7 | 21 | 365 | 385 |
| GAEF01002199.1 | 1 | p1 | (A)10 | 10 | 62 | 71 |
| GAEF01002342.1 | 1 | p3 | (GCC)5 | 15 | 473 | 487 |
| GAEF01002342.1 | 2 | p1 | (T)10 | 10 | 965 | 974 |
| GAEF01013419.1 | 1 | p1 | (T)22 | 22 | 1 | 22 |
| GAEF01022475.1 | 1 | p3 | (AAG)5 | 15 | 558 | 572 |
| GAEF01027630.1 | 1 | p3 | (GCA)6 | 18 | 96 | 113 |
| GAEF01027630.1 | 2 | p4 | (TGGT)5 | 20 | 454 | 473 |
| GAEF01032367.1 | 1 | p1 | (A)13 | 13 | 183 | 195 |
| GAEF01033036.1 | 1 | p2 | (TC)6 | 12 | 70 | 81 |
| GAEF01043433.1 | 1 | p3 | (AGC)5 | 15 | 127 | 141 |
| GAEF01066815.1 | 1 | p1 | (C)16 | 16 | 7 | 22 |
| GAEF01082111.1 | 1 | p2 | (AG)7 | 14 | 323 | 336 |
| JP931192.1 | 1 | p1 | (A)29 | 29 | 342 | 370 |
| JP907678.1 | 1 | p1 | (T)62 | 62 | 20 | 81 |
| JP896743.1 | 1 | p3 | (TGG)5 | 15 | 236 | 250 |
| JP896743.1 | 2 | c | (A)18g(A)16 | 35 | 1020 | 1054 |
| JP894741.1 | 1 | p1 | (T)40 | 40 | 18 | 57 |
| JP876391.1 | 1 | p1 | (T)15 | 15 | 744 | 758 |
| JP875212.1 | 1 | c | (A)27n(A)15n(A)14n(A)11 | 70 | 1467 | 1536 |
| JP870688.1 | 1 | c | (A)12n(A)11 | 24 | 1443 | 1466 |
| JP866401.1 | 1 | p1 | (T)14 | 14 | 34 | 47 |
| JP851192.1 | 1 | p3 | (GAA)5 | 15 | 303 | 317 |
| JP827398.1 | 1 | c | (A)33n(A)11 | 45 | 648 | 692 |
| JP822941.1 | 1 | c | (T)15n(T)13 | 29 | 13 | 41 |
| JW032020.1 | 1 | p1 | (A)12 | 12 | 230 | 241 |
| JW031841.1 | 1 | p3 | (AGC)6 | 18 | 570 | 587 |
| JV991710.1 | 1 | p3 | (CCG)5 | 15 | 33 | 47 |
| JV987837.1 | 1 | p3 | (CCG)5 | 15 | 535 | 549 |
| JV944561.1 | 1 | p1 | (T)10 | 10 | 307 | 316 |
| JV942652.1 | 1 | p1 | (A)14 | 14 | 44 | 57 |
| JV924190.1 | 1 | p1 | (A)10 | 10 | 109 | 118 |
| JV908614.1 | 1 | p1 | (T)11 | 11 | 1 | 11 |
| JV908248.1 | 1 | p2 | (GA)16 | 32 | 582 | 613 |
| JV905457.1 | 1 | p2 | (GA)16 | 32 | 582 | 613 |
| JV903614.1 | 1 | p1 | (A)12 | 12 | 710 | 721 |
| JV887791.1 | 1 | p2 | (CA)8 | 16 | 112 | 127 |
| JV886512.1 | 1 | p3 | (CAC)8 | 24 | 228 | 251 |
| JV886512.1 | 2 | p3 | (AAG)17 | 51 | 1325 | 1375 |
| JV883062.1 | 1 | p1 | (T)10 | 10 | 486 | 495 |
| JV872689.1 | 1 | p3 | (GAG)8 | 24 | 29 | 52 |
| JV872329.1 | 1 | p3 | (GCG)5 | 15 | 1678 | 1692 |
| JV870192.1 | 1 | p3 | (CGA)6 | 18 | 17 | 34 |
| JV866930.1 | 1 | p1 | (G)11 | 11 | 2150 | 2160 |
| JV863864.1 | 1 | p2 | (TG)12 | 24 | 2310 | 2333 |
| JV863140.1 | 1 | p1 | (T)11 | 11 | 1 | 11 |
| JV842032.1 | 1 | p1 | (A)10 | 10 | 5 | 14 |
| JV814540.1 | 1 | p1 | (A)10 | 10 | 172 | 181 |
| JP232032.1 | 1 | p2 | (TC)6 | 12 | 16 | 27 |
| JP217907.1 | 1 | c | (TA)8tgtgtaatagcattatcaaggagaacactattcaaacct(A)15 | 70 | 1290 | 1359 |
| JP216817.1 | 1 | p1 | (T)10 | 10 | 389 | 398 |
| JP211167.1 | 1 | p1 | (A)11 | 11 | 16 | 26 |
| HP628784.1 | 1 | p2 | (AC)6 | 12 | 358 | 369 |
| HP609379.1 | 1 | p1 | (C)11 | 11 | 1020 | 1030 |
| HP609361.1 | 1 | p1 | (C)11 | 11 | 1020 | 1030 |
| HP609361.1 | 2 | p3 | (CTT)6 | 18 | 2957 | 2974 |
| HP609353.1 | 1 | p1 | (C)11 | 11 | 1020 | 1030 |
| HP609344.1 | 1 | p1 | (C)11 | 11 | 1020 | 1030 |
| HP609339.1 | 1 | p1 | (C)11 | 11 | 1020 | 1030 |

**Supplementary Table 2.** Details of template protein used in structure prediction in Phyre2

| Description | Hit (Template PDB ID) | Confidence (%) | | Sequence identity (%) | Alignment coverage (%) | Resolution | Hit info 1 | Hit info 2 | Hit info 3 |
| --- | --- | --- | --- | --- | --- | --- | --- | --- | --- |
| JP928422.1 | 3APO | 100 | 26 | | 63 | 2.4 | PDB header:oxidoreductase | PDBTitle: crystal structure of full-length erdj5 | Chain: A: PDB Molecule:dnaj homolog subfamily c member 10; |
| JP879666.1 | 4J80 | 99.9 | 28 | | 20 | 2.9 | PDB header:chaperone | PDBTitle: thermus thermophilus dnaj | Chain: B: PDB Molecule:chaperone protein dnaj 2; |
| CV762197.1 | 2KHO | 99.9 | 50 | | 69 | UNK | PDB header:chaperone | PDBTitle: nmr-rdc / xray structure of e. coli hsp70 (dnak) chaperone (1-605)2 complexed with adp and substrate | Chain: A: PDB Molecule:heat shock protein 70; |
| CJ542498.1 | 6IAC | 29 | 50 | | 13 | 3.9 | PDB header:structural protein | PDBTitle: portal and tail of native bacteriophage p68 | Chain: L: PDB Molecule:inner core protein; |
| GD186945.1 | 6OB7 | 98.8 | 19 | | 94 | 2.3 | PDB header:transport protein | PDBTitle: human equilibrative nucleoside transporter-1, dilazep bound | Chain: A: PDB Molecule:equilibrative nucleoside transporter 1; |
| HG916218.1 | 2BPT | 65.2 | 61 | | 17 | 1.99 | PDB header:nuclear transport | PDBTitle: structure of the nup1p:kap95p complex | Chain: B: PDB Molecule:nucleoporin nup1; |
